# Unlocking Plant Resilience: Metabolomic Insights into Abiotic Stress Tolerance in Crops

**DOI:** 10.3390/metabo15060384

**Published:** 2025-06-09

**Authors:** Agata Głuchowska, Bartłomiej Zieniuk, Magdalena Pawełkowicz

**Affiliations:** 1 Department of Plant Genetics, Breeding and Biotechnology, Institute of Biology, Warsaw University of Life Sciences-SGGW, 159 Nowoursynowska Str., 02-776 Warsaw, Poland; 2 Department of Chemistry, Institute of Food Sciences, Warsaw University of Life Sciences-SGGW, 159C Nowoursynowska Str., 02-776 Warsaw, Poland

**Keywords:** crop resilience, climate-smart agriculture, abiotic stress tolerance, metabolomics, multi-omics integration, omics technologies, metabolic profiling, marker-assisted selection (MAS), genomic selection (GS), plant adaptation mechanisms

## Abstract

**Background/Objectives:** In the context of accelerating climate change and growing food insecurity, improving crop resilience to abiotic stresses such as drought, salinity, heat, and cold is a critical agricultural and scientific challenge. Understanding the biochemical mechanisms that underlie plant stress responses is essential for developing resilient crop varieties This review aims to provide an integrative overview of how metabolomics can elucidate biochemical mechanisms underlying stress tolerance and guide the development of stress-resilient crops. **Methods:** We reviewed the recent literature on metabolomic studies addressing abiotic stress responses in various crop species, focusing on both targeted and untargeted approaches using platforms such as nuclear magnetic resonance (NMR), liquid chromatography–mass spectrometry (LC-MS), and gas chromatography–mass spectrometry (GC-MS). We also included emerging techniques such as capillary electrophoresis–mass spectrometry (CE-MS), ion mobility spectrometry (IMS-MS), Fourier transform infrared spectroscopy (FT-IR), and data-independent acquisition (DIA). Additionally, we discuss the integration of metabolomics with transcriptomics and physiological data to support system-level insights. **Results:** The reviewed studies identify common stress-responsive metabolites, including osmoprotectants, antioxidants, and signaling compounds, which are consistently linked to enhanced tolerance. Novel metabolic biomarkers and putative regulatory hubs are highlighted as potential targets for molecular breeding and bioengineering. We also address ongoing challenges related to data standardization and reproducibility across analytical platforms. **Conclusions:** Metabolomics is a valuable tool for advancing our understanding of plant abiotic stress responses. Its integration with other omics approaches and phenotypic analyses offers promising avenues for improving crop resilience and developing climate-adaptive agricultural strategies.

## 1. Introduction

Climate change is increasing the frequency and severity of abiotic stresses—such as drought, salinity, extreme temperatures, and oxidative damage—that negatively impact global agricultural productivity [1]. These environmental challenges threaten food security by hindering crop growth, lowering yields, and compromising nutritional quality [1]. As sessile organisms, plants have developed complex biochemical and physiological mechanisms to perceive and respond to these stresses, allowing them to adapt to fluctuating environmental conditions [1]. Understanding these adaptive responses is essential for creating strategies to enhance crop resilience and ensure sustainable food production [1].

Metabolomics, the comprehensive analysis of metabolites within a biological system, has become a powerful tool for investigating plant responses to abiotic stresses. Metabolites—small molecules involved in various cellular processes—act as direct indicators of physiological states and can reflect the cumulative effects of genetic, environmental, and epigenetic factors [2,3]. By profiling the metabolome, researchers can gain insights into the dynamic changes occurring within plants under stress conditions, facilitating the identification of biomarkers associated with stress tolerance [2]. This approach bridges the gap between genotype and phenotype, providing a system-level understanding of plant stress biology [4].

The application of metabolomics in plant research has advanced significantly due to the development of sophisticated analytical techniques. Gas chromatography–mass spectrometry (GC-MS), liquid chromatography–mass spectrometry (LC-MS), and nuclear magnetic resonance (NMR) spectroscopy rank among the most widely used platforms [4,5]. These technologies provide high sensitivity, resolution, and reproducibility, enabling the detection and quantification of a broad spectrum of metabolites [1,5]. GC-MS is especially effective for analyzing volatile and thermally stable metabolites, while LC-MS offers versatility in separating and identifying a wide range of metabolites. NMR spectroscopy delivers detailed structural information about metabolites [5]. The integration of these analytical techniques has fostered a more holistic understanding of plant metabolism, revealing how plants reprogram their metabolic pathways to cope with abiotic stresses [6].

**Figure 1:**
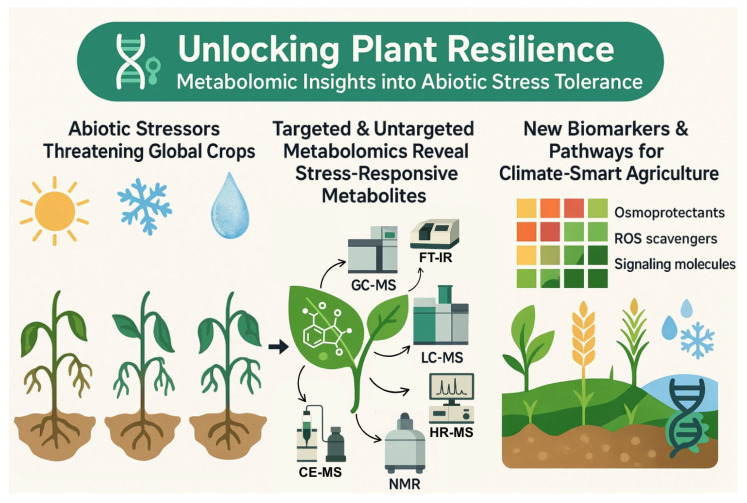
An overview of metabolomic insights into abiotic stress tolerance.

The goal of this article is to explore the role of metabolomics in understanding plant responses to abiotic stresses, highlighting the importance of advanced analytical techniques in this field. By examining recent research and technological advancements, we aim to emphasize the potential of metabolomics in contributing to the development of climate-resilient crops (Figure 1).

## 2. Metabolomics in Plant Research

Metabolomics is a powerful tool for studying the biochemical composition of plants and their responses to various biotic and abiotic stresses. It is a rapidly evolving field that focuses on the comprehensive study of metabolites, which are small molecules involved in metabolic pathways. The characterization of plant metabolism through metabolomic approaches is crucial for understanding plant physiology, stress responses, and biochemical adaptations.

### 2.1. Methods Review

Among the many analytical techniques used for metabolomic studies, gas chromatography–mass spectrometry (GC-MS), liquid chromatography–mass spectrometry (LC-MS), and nuclear magnetic resonance (NMR) spectroscopy are the most widely adopted. Each of these platforms offers unique advantages and faces specific challenges when applied to plant metabolomics.

#### 2.1.1. Gas Chromatography–Mass Spectrometry (GC-MS)

GC-MS is one of the most commonly utilized methods for metabolite analysis, particularly for volatile and thermally stable compounds. This technique provides high sensitivity and reproducibility, making it ideal for analyzing primary metabolites such as sugars, amino acids, and organic acids, which are often involved in essential metabolic processes. GC-MS also benefits from extensive spectral libraries that aid in the identification of metabolites with high precision [7].

However, GC-MS has limitations, especially when analyzing high-molecular-weight or thermally labile metabolites, such as lipids, nucleotides, and certain secondary metabolites. Additionally, this technique often requires chemical derivatization of non-volatile or unstable compounds, which can introduce variability and complexity in sample preparation [8,9]. Despite these drawbacks, GC-MS remains a cornerstone in metabolomic analyses, particularly for profiling volatile organic compounds (VOCs) and secondary metabolites that can be derivatized.

#### 2.1.2. Liquid Chromatography–Mass Spectrometry (LC-MS)

LC-MS is highly versatile and capable of analyzing a broad spectrum of metabolites, including both primary and secondary ones. It is particularly effective for polar and non-polar compounds that cannot be analyzed directly by GC-MS due to their volatility or thermal instability [10,11,12]. LC-MS offers excellent sensitivity and high-throughput analysis, making it suitable for large-scale studies of plant metabolites. This method is well suited for detecting low-abundance metabolites, which are often critical in studying plant responses to environmental stress.

One of the main advantages of LC-MS is that it does not require derivatization, simplifying the sample preparation process and minimizing sample loss. However, LC-MS is prone to ion suppression effects, where the presence of other components in the sample matrix can interfere with the ionization of target metabolites, leading to inaccuracies in quantification [12]. Despite this, LC-MS remains an essential tool in metabolomics, offering the ability to capture a broader range of metabolites compared to GC-MS.

#### 2.1.3. Nuclear Magnetic Resonance (NMR) Spectroscopy

NMR spectroscopy is a non-destructive, highly reproducible method that provides both quantitative and structural information about metabolites. It is especially valuable for its ability to elucidate the molecular structure of compounds without the need for external standards, making it a powerful tool for discovering unknown metabolites [13]. NMR spectroscopy is also highly reproducible and allows for the analysis of intact plant tissues, as well as other complex samples, such as cell cultures and leaves, with minimal preparation.

However, NMR has limitations, primarily in its sensitivity compared to MS-based techniques. It typically requires larger quantities of metabolites to detect signals at meaningful levels, which can limit the analysis of low-abundance compounds. Additionally, the high cost of NMR instrumentation and its relatively slow data acquisition process make it less suitable for high-throughput studies when compared to LC-MS [14,15]. Despite these drawbacks, NMR remains an invaluable tool for plant metabolomics due to its capacity to provide detailed molecular insights without the need for destructive analysis.

#### 2.1.4. Comparison

Each of these techniques—GC-MS, LC-MS, and NMR—has distinct strengths that make them suitable for different applications in plant metabolomics. GC-MS is ideal for analyzing volatile and derivatized metabolites, while LC-MS excels at profiling a wide range of metabolites, including both polar and non-polar compounds. NMR is invaluable for providing structural and quantitative information in a non-destructive manner. Often, a combination of these techniques is used to deliver a comprehensive view of plant metabolomes, overcoming the limitations of each individual method (Table 1).

### 2.2. Sample Preparation and Extraction Methods

Effective sample preparation directly affects the accuracy, reproducibility, and completeness of metabolite profiling, making it fundamentally important in plant metabolomics (Table 2). The choice of extraction methods and solvents should be customized to fit the specific analytical platform—GC-MS, LC-MS, or NMR—to ensure optimal recovery of metabolites [16,17,18].

Plant metabolism is highly dynamic and can be quickly altered by mechanical injury or environmental changes during harvest. Immediate quenching of metabolic activity is essential for preserving the native state of the metabolome. Flash freezing with liquid nitrogen remains the gold standard due to its rapid cooling rate [18]. Cold methanol or dry ice–ethanol baths serve as alternatives. They are particularly useful under field conditions, albeit with slightly lower preservation fidelity [19]. Homogenization is typically performed under cryogenic conditions to prevent thawing and degradation. Grinding with a mortar and pestle in liquid nitrogen is common in small-scale studies, whereas bead-based homogenizers are favored for their efficiency in high-throughput workflows [18].

No single extraction method is universally ideal because the metabolome encompasses a variety of chemical properties. Polar metabolites are typically extracted using methanol, ethanol, and water mixtures [20]. Non-polar metabolites, such as lipids, may require solvents like chloroform, although its use in LC-MS is limited [19]. Biphasic systems such as methanol:chloroform:water, are widely employed for broader metabolite recovery [18]. Advanced methods, such as ultrasound-assisted and microwave-assisted extraction, are gaining popularity due to their improved efficiency and reduced solvent consumption [21]. GC-MS necessitates that metabolites be volatile and thermally stable, which requires derivatization with agents like MSTFA (*N*-Methyl-*N*-(trimethylsilyl)trifluoroacetamide) that react with -OH, -COOH, -NH, and -SH groups, enhancing compound volatility and thermal stability [20]. LC-MS accommodates a wide range of metabolites with minimal sample preparation. Simple methanol or acetonitrile extractions are common, and clean-up steps, such as filtration or SPE, are often used to reduce matrix effects [5]. NMR spectroscopy, while less sensitive, demands minimal sample preparation. Deuterated solvents, such as D_2_O and CD_3_OD, are utilized, and pH must be controlled to avoid shifts in the chemical signal [21,22]. Internal standards, such as TSP, assist in quantification and referencing.

Quality control is essential for the reliability of metabolomics. QC samples, internal standards (often isotopically labeled), and detailed Standard Operating Procedures (SOPs) are key components of robust analytical workflows [5,17]. In this context, SOPs are comprehensive, written instructions designed to achieve uniformity and consistency in performing of specific tasks or processes within an organization or research setting. SOPs are employed to ensure that procedures are consistently followed, helping to maintain quality control, reduce errors, and ensure reproducibility in analytical workflows.

In summary, tailored extraction strategies and rigorous sample handling protocols are critical in plant metabolomics. Platform-specific optimizations ensure reproducible and comprehensive metabolite profiling.

### 2.3. Expanding the Metabolomic Toolbox: Novel Approaches to Abiotic Stress Analysis

Recent advances in analytical technologies have significantly expanded the toolkit available for investigating metabolomic responses of plants to abiotic stress, offering improved resolution, sensitivity, and chemical coverage. Among these, capillary electrophoresis–mass spectrometry (CE-MS) has emerged as a valuable technique due to its high separation efficiency for charged and polar metabolites. Its minimal sample requirement and compatibility with aqueous matrices make it particularly suitable for profiling metabolites involved in stress signaling and defense pathways [23,24].

Ion mobility mass spectrometry (IMS-MS), by incorporating an additional ion mobility separation step, enhances the structural resolution of complex metabolomes and improves discrimination of isomeric compounds, thereby facilitating more detailed characterization of plant responses to stress [25,26].

Another complementary approach, Fourier transform infrared spectroscopy (FT-IR), enables rapid, non-destructive chemical analysis of plant tissues by detecting functional group vibrations, thus offering a useful snapshot of macromolecular changes induced by environmental stressors [27].

High-resolution mass spectrometry (HR-MS) further strengthens metabolomic investigations through its high mass accuracy and resolution, allowing for the confident identification of stress-related metabolites even at low concentrations [28]. Moreover, data-independent acquisition (DIA) techniques in mass spectrometry have transformed quantitative metabolomics by enabling comprehensive and unbiased fragmentation of all detectable ions within a given mass range. This strategy improves reproducibility and enables robust comparisons across multiple stress conditions or genotypes [29]. Collectively, these cutting-edge technologies provide a more holistic and precise understanding of how crops adapt their metabolism to withstand abiotic stress, and their integration into future studies will be essential for advancing crop resilience research.

While nuclear magnetic resonance (NMR), gas chromatography–mass spectrometry (GC-MS), and liquid chromatography–mass spectrometry (LC-MS) remain foundational platforms in plant metabolomics due to their robustness, reproducibility, and established protocols, the integration of emerging techniques such as CE-MS, IMS-MS, FT-IR, HR-MS, and DIA provides substantial complementary advantages. Traditional methods offer broad applicability and have been instrumental in identifying key metabolic markers under abiotic stress. However, they are sometimes limited by factors such as lower sensitivity (in the case of NMR), or reduced ability to resolve structurally similar compounds (in both GC-MS and LC-MS). In contrast, newer techniques enhance metabolite coverage, improve structural resolution, and allow for higher-throughput, non-targeted analysis. Thus, rather than replacing established methodologies, these advanced approaches should be viewed as synergistic tools that expand the analytical landscape. Incorporating them into multi-platform metabolomic workflows promises to yield deeper, more nuanced insights into the biochemical networks that underpin plant stress resilience.

### 2.4. Standardization and Reproducibility in Metabolomics Across Analytical Platforms

Standardization and reproducibility remain pivotal challenges in metabolomics, particularly when comparing data across different analytical platforms such as gas chromatography–mass spectrometry (GC-MS) and liquid chromatography–mass spectrometry (LC-MS). GC-MS benefits from a high degree of standardization, largely due to the consistent use of electron ionization (EI) at 70 eV, which facilitates reproducible mass spectra and the development of extensive spectral libraries like NIST and the Golm Metabolome Database. This consistency enhances inter-laboratory comparability and data reproducibility [30,31].

In contrast, LC-MS faces greater variability stemming from differences in ionization techniques (e.g., electrospray ionization vs. atmospheric pressure chemical ionization), chromatographic conditions, and instrument configurations. These variations can lead to significant discrepancies in metabolite detection and quantification, posing challenges for data standardization. Moreover, LC-MS is susceptible to matrix effects such as ion suppression, which can compromise analytical accuracy and reproducibility [15,32].

To address these challenges, the metabolomics community has initiated efforts to develop standardized protocols and reporting guidelines. The Metabolomics Standards Initiative (MSI), for example, has proposed minimum reporting standards to enhance data transparency and reproducibility [33,34]. Additionally, the use of internal standards, quality control samples, and inter-laboratory comparison studies is recommended to improve data reliability across platforms. Despite these advancements, achieving comprehensive standardization remains an ongoing endeavor, necessitating continued collaboration and methodological refinement within the field [35,36].

### 2.5. Analytical Approaches: Targeted vs. Untargeted Metabolomics

The characterization of plant metabolism through metabolomic approaches is vital for understanding plant physiology, stress responses, and biochemical adaptations. The two main strategies in metabolomics are targeted and untargeted approaches, each offering unique insights into the plant metabolome. These strategies vary in their goals, methodologies, and applications, and both provide essential information for plant research.

#### 2.5.1. Targeted Metabolomics

Targeted metabolomics involves the quantification and analysis of a pre-defined set of metabolites, often grounded in specific hypotheses about metabolic pathways. This approach is highly selective, aiming to measure metabolites of interest that are thought to contribute to certain biochemical processes or responses. The analytical techniques employed in targeted metabolomics, such as LC-MS and GC-MS, are optimized for high sensitivity and accuracy in quantifying known compounds [37].

One of the main advantages of targeted metabolomics is its high reproducibility and precision, as it relies on a known set of metabolites and requires fewer analytical steps than untargeted approaches. This facilitates a focused study of specific metabolic pathways, such as those involved in secondary metabolism or stress response, and enables the correlation of metabolite changes with phenotypic traits [38]. Targeted approaches have been extensively used to examine specific classes of plant metabolites, including flavonoids, phenolic acids, alkaloids, and terpenoids [39].

However, the primary limitation of targeted metabolomics is its narrow focus, as it misses the broader picture of the metabolome. The approach is constrained by prior knowledge, meaning that any metabolites outside the targeted list will not be detected, potentially overlooking novel biomarkers or pathways of interest [40].

#### 2.5.2. Untargeted Metabolomics

Untargeted metabolomics, on the other hand, aims to capture as many metabolites as possible in a given biological sample without prior knowledge of their identities. This approach is typically used to explore the entire metabolome, providing a holistic view of the metabolic state of the organism under study. Analytical techniques such as high-resolution LC-MS, GC-MS, and NMR spectroscopy are employed to generate large datasets that can be subjected to multivariate statistical analyses [41].

The primary advantage of untargeted metabolomics is its ability to detect a wide array of metabolites, including those not previously identified or studied, and to uncover unexpected metabolic changes. This approach has been instrumental in identifying novel biomarkers for stress tolerance, disease resistance, and growth conditions in plants [42]. Additionally, untargeted analysis enables the discovery of new metabolic pathways, which can provide deeper insights into plant biology and lead to the identification of new therapeutic targets [43].

However, untargeted metabolomics also presents significant challenges. The vast amount of data generated can be overwhelming, requiring advanced computational tools and statistical methods to identify meaningful patterns. Furthermore, the identification of unknown metabolites remains a complex task due to the lack of reference standards for many compounds, making it difficult to establish accurate quantifications and identifications [44].

#### 2.5.3. Complementarity of Targeted and Untargeted Approaches

Both targeted and untargeted metabolomics possess their distinct strengths and weaknesses. In many instances, combining both strategies yields the most comprehensive insights into plant metabolism. Targeted metabolomics can confirm specific hypotheses regarding metabolic changes in response to environmental stressors or genetic modifications, while untargeted metabolomics offers a broader understanding of the metabolic shifts occurring within the plant. Recent advancements in data integration enable researchers to merge the strengths of both approaches, resulting in more robust conclusions in plant metabolomics research [45].

In summary, targeted and untargeted metabolomics are two complementary approaches that, when utilized together, offer a deeper and more accurate understanding of plant metabolic networks. Targeted metabolomics is ideal for hypothesis-driven studies focusing on specific metabolites, while untargeted metabolomics facilitates the comprehensive discovery of previously unidentified metabolic compounds. As both technologies evolve, integrating these approaches holds significant potential for enhancing our understanding of plant biology and improving crop resilience and productivity.

## 3. Plant Metabolic Responses and the Critical Role of Secondary Metabolites to Abiotic Stresses

Abiotic stresses, including drought, salinity, extreme temperatures, heavy metal contamination, and nutrient deficiencies, significantly hinder global agriculture, reducing crop yields by over 60% and posing serious challenges for plant breeding efforts. The United Nations projects that the global population will increase to 8.5 billion by 2030 and 9.7 billion by 2050, further exerting pressure on agricultural systems [46].

Plants employ an advanced metabolic toolkit to address these challenges. This includes the accumulation of osmolytes, such as proline and glycine betaine (trimethylglycine), for osmotic balance and stress-specific secondary metabolites, like phenolics, terpenoids, and alkaloids, for scavenging ROS and signaling. Additionally, molecular chaperones, including heat shock proteins, help maintain protein integrity These responses are coordinated through dynamic phytohormone networks, such as ABA and jasmonates, along with epigenetic changes that enhance stress memory and resource reallocation strategies that prioritize survival over growth.

### 3.1. Salinity Stress

Salinity critically impairs crop growth from germination to harvest, increasingly threatening agricultural productivity in arid and semiarid regions with low rainfall and high evaporation. Currently affecting over 7% of global land and nearly 20% of arable land, salt-degraded land expands by 1 to 2% per year. Projections suggest over 50% of arable land could become unproductive by 2050 due to salt stress [47].

Soil salinization occurs when water-soluble salts (Na^+^, Cl^−^, K^+^, SO_4_^2−^) accumulate in the root zone. This accumulation disrupts the osmotic balance, making it challenging for plants to absorb water and causing hyperionic stress. Excess Na^+^ and Cl^-^ are the primary contributors, with high Na^+^ levels resulting in soil sodicity. Most modern crops, especially glycophytes like tomatoes and rice, struggle to grow in saline soils, unlike halophytes, which thrive in such conditions. The impact of salinity on plants occurs in two stages: the first involves osmotic stress, leading to reduced water potential and stunted growth, while the second stage entails prolonged ionic toxicity due to Na^+^/Cl^−^ buildup, nutrient imbalance, and oxidative damage. The consequences include stunted growth, chlorosis, impaired photosynthesis, electrolyte leakage, and significant setbacks during the reproductive stage, along with the overproduction of reactive oxygen species (ROS) in chloroplasts [48,49].

**Figure 2:**
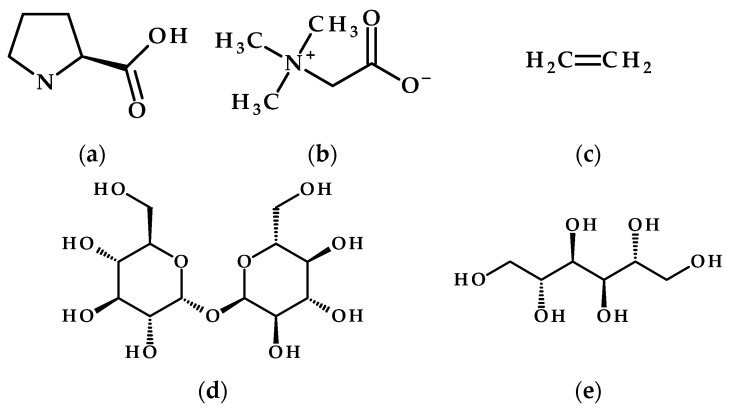
Chemical structures of (**a**) proline, (**b**) glycine betaine, (**c**) ethylene, (**d**) trehalose, and (**e**) mannitol.

Proline (the only proteinogenic amino acid that is a secondary amine; Figure 2a) serves as a crucial compatible osmolyte and antioxidant, increasing in response to salt stress, which helps plants maintain cell turgor. The elevated proline levels have been recognized and utilized as a physiological indicator of how plants react to salinity stress [48]. When applied as an exogenous compound in salt-stressed crops such as *Cucumis sativus*, *C. melo*, *Helianthus annus*, *Nicotiana tabacum*, *Olea europaea*, *Oryza sativa*, *Triticum durum*, and *Zea mays*, foliar application of proline increased plant growth, positively impacted yield characteristics, and mitigated salt-related damage by enhancing antioxidants (SOD, CAT, APX) and reducing oxidative markers [47].

Furthermore, salt stress can affect the expression of essential enzymes involved in proline metabolism, including pyrroline-5-carboxylate synthetase (P5CS), pyrroline-5-carboxylate reductase (P5CR), and proline dehydrogenase (PDH) [50]. High proline accumulation has also been observed in salt-tolerant varieties of green gram (*Phaseolus aureus*), potato (*Solanum tuberosum*), glasswort (*Salicornia europaea*), rice (*O. sativa*), and seashore mallow (*Kosteletzkya virginica*) [49,51].

Glycine betaine, another compound involved in osmoregulation, is a type of quaternary ammonium compound (Figure 2b) that increases in concentration in various plants, such as common bean (*P. vulgaris*), potato (*S. tuberosum*), and maize (*Z. mays*), when they experience dehydration and salt stress [48,52,53,54]. This compound is produced from choline in chloroplasts and primarily functions to stabilize proteins and membranes, scavenge reactive oxygen species (ROS), and maintain osmotic balance. Numerous studies indicate that the external application of this quaternary ammonium compound enhances growth, regulates ion homeostasis (decreasing Na^+^ levels and increasing K^+^ levels), and boosts antioxidant activity [54,55].

In potatoes, glycine betaine alleviates salinity stress through various mechanisms. Applying externally through foliar sprays or root treatments decreases Na^+^ accumulation, maintains K^+^/Na^+^ balance, and boosts antioxidant enzymes (SOD, CAT), which helps reduce oxidative damage. Additionally, glycine betaine protects chlorophyll and enhances photosynthetic efficiency. Transgenic potatoes that overexpress BADH (Betaine-Aldehyde Dehydrogenase) from spinach or barley can accumulate higher levels of this compound, promote growth and minimize yield loss under saline conditions. Moreover, it works synergistically with other osmolytes, such as proline and mannitol [52].

Similar observations were also noted in common bean (*P. vulgaris*) under NaCl stress. Glycine betaine (25 mM) significantly decreased Na^+^ accumulation and enhanced K^+^ uptake, while also reducing oxidative damage by lowering lipid peroxidation and electrolyte leakage. Ultimately, plants treated with this compound exhibited restored photosynthetic pigments, improved membrane stability, and a 29.8% to 59.4% increase in pod yield under saline conditions, showcasing its effectiveness in enhancing crop resilience in salty environments [54].

Ethylene (Figure 2c), the first gaseous plant hormone, is crucial for regulating plant growth and development. A review [56] highlights ethylene’s vital role in managing plant salinity stress tolerance by maintaining ion homeostasis (Na^+^/K^+^ balance), boosting antioxidant defenses, and facilitating hormonal interactions. Ethylene signaling promotes salinity tolerance by activating EIN3/EIL1 transcription factors, which improve photosynthesis, reduce reactive oxygen species (ROS) through both enzymatic and non-enzymatic antioxidants, and regulate stress-responsive genes. It works synergistically or antagonistically with hormones such as ABA, JA, and auxin to optimize growth and adaptation to stress, while also impacting seed germination and decreasing programmed cell death [56].

Sugars and polyols are crucial for plants’ ability to withstand salt stress, as they safeguard photosynthetic structures and facilitate osmotic adjustment through the accumulation of osmolytes, like proline and glycine betaine. Although trehalose (Figure 2d) is not a primary osmoprotectant in many plants, trehalose-6-phosphate (T6P) acts as a sensor for sucrose availability, connecting environmental stress responses to developmental processes. On a molecular level, trehalose activates antioxidant enzymes (SOD, CAT, APX), enhance the expression of stress-responsive genes (NHX1, SOS1) and boost ABA-mediated signaling, which helps mitigate oxidative damage and ion toxicity. Fine-tuning trehalose metabolism by precisely targeting its biosynthesis (TPS/TPP enzymes) or hydrolysis (trehalase) has proven effective in improving resilience to abiotic stress [57]. This has been shown in transgenic crops, like rice and tomatoes, which demonstrate enhanced salt tolerance. Nonetheless, future research should concentrate on refining application methods and exploring interactions with hormonal and metabolic networks to maximize the agricultural benefits of trehalose, while also preventing pleiotropic effects, such as those linked with stomatal-specific trehalase overexpression for drought tolerance, without compromising growth [58].

In the case of mannitol (Figure 2e), ref. [59] showed that pretreating wheat grains with trehalose and mannitol (by soaking in a 10 mM sugar/polyol solution) improves the resilience of wheat to salt stress through enhancements in both enzymatic and non-enzymatic antioxidant pathways. The authors observed an increase in antioxidant enzymes (APX and CAT) and phenolic compounds, alongside a reduction in lipid peroxidation (MDA levels) under 150 mM NaCl stress [59]. Additionally, introducing the *E. coli* mtlD gene into indica rice via *Agrobacterium*-mediated transformation facilitated mannitol biosynthesis. Molecular analyses confirmed the integration of transgenes, showing that transgenic lines accumulated mannitol and demonstrated superior seed germination and growth under salinity stresses (150 mM NaCl) compared to wild-type plants. This implies that the low levels of mannitol in transgenic rice bolster stress resilience, likely by aiding osmotic adjustment or enhancing antioxidant activities, thereby presenting a promising strategy for improving crops in adverse conditions [60].

In conclusion, salinity stress, caused by soil salt accumulation, disrupts osmotic balance and induces ionic toxicity, negatively affecting crop growth, photosynthesis, and yield, particularly in arid regions. Plants reduce salt damage through osmolytes (proline, glycine betaine) and hormones (ethylene) that help regulate ion homeostasis, boost antioxidant defenses, and stabilize cellular structures. Furthermore, the external application or genetic engineering of stress-responsive compounds like proline, trehalose, and mannitol enhances salt tolerance in crops such as rice, wheat, and potatoes, providing strategies to maintain productivity in saline environments.

### 3.2. Drought Stress

Drought and water scarcity present major challenges. In the early 21st century, 79 cities faced severe droughts, and by 2050, as many as 2.37 billion urban residents (35–51% of the global urban population) might encounter water scarcity [61]. Drought considerably diminishes crop yields (by 50–70%), disrupting physiological processes such as photosynthesis, nutrient absorption, and the accumulation of ROS, which leads to oxidative stress. Key crops, including maize, wheat, rice, and cotton, are especially susceptible to notable yield reductions [62].

Phytohormones such as abscisic acid (ABA), auxin, cytokinins (CK), gibberellins (GA), jasmonates (JA), salicylic acid (SA), ethylene (ET), brassinosteroids (BR), and strigolactones (SL) play a crucial role in regulating drought responses by influencing stomatal closure, root architecture, ROS scavenging, and the expression of stress-responsive genes. ABA is essential for regulating stomatal function and osmotic adjustments, while auxin aids in root development and maintaining ROS levels. CKs and GA have opposing effects: CK inhibition enhances drought resilience, whereas a decrease in GA (through catabolic genes like GA2ox) mitigates growth-related weaknesses. JA and SA collaborate with ABA to strengthen antioxidant defenses and improve systemic resistance. Ethylene and BRs promote stress resilience by activating transcription factors (such as ERFs) and managing ROS, while SLs are involved in regulating stomatal closure and mediating root-to-shoot communication [63].

Under drought stress, many secondary metabolites are essential for helping plants adapt and survive. These chemical compounds play a significant role in protecting plants against abiotic stress, and their accumulation enhances a plant’s resilience. Plants produce various types of secondary metabolite to safeguard and regulate their functions under different stress conditions. According to [62], they are classified into three primary categories: (a) phenolic compounds (including phenolic acids, lignin, coumarins, stilbenes, lignans, flavonoids, and tannins), (b) terpene derivatives (such as carotenoids, plant volatiles, and sterols), and (c) nitrogen-containing compounds, including glucosinolates and alkaloids [62].

Phenolic compounds are a diverse group of organic molecules found in plants, characterized by aromatic rings with hydroxyl groups. They exist in fruits, vegetables, tea, coffee, and cereals. Known for their antioxidant properties, these compounds neutralize harmful free radicals and reduce oxidative stress, potentially providing anti-inflammatory and disease prevention benefits. Their stability and bioactive characteristics have led to extensive research in food preservation, nutraceuticals, and cosmetics. In plants, they are crucial for regulating growth, reproduction, and defense against microorganisms and environmental stress. Additionally, they act as signaling molecules that initiate plant–microbe symbioses, such as arbuscular mycorrhiza and legume–rhizobium relationships [64].

**Figure 3:**
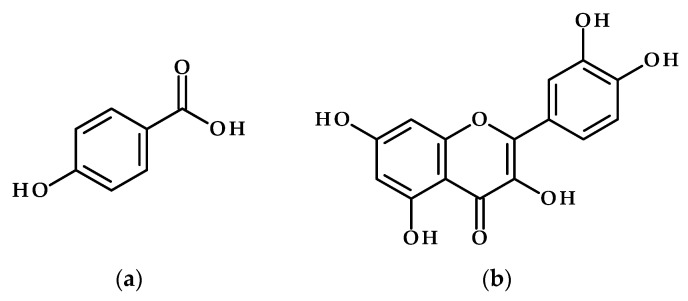
Core chemical structures of phenolic acids and flavonoids involved in plant drought stress responses, i.e., (**a**) *p*-hydroxybenzoic acid and (**b**) quercetin.

It has been confirmed that drought-resistant rice varieties accumulate higher amounts of total phenolics, flavonoids, and antioxidants, which are linked to improved drought resistance. This is exemplified by the tolerant cultivar Q8 compared to the susceptible Q2. Notably, specific phenolic acids, such as vanillic and *p*-hydroxybenzoic acids (Figure 3a), exhibited significant increases only in Q8 under drought stress, indicating their essential role in reducing oxidative damage [65].

These results highlight the potential for leveraging targeted phenolic compounds to enhance rice’s resilience to water scarcity through methods like metabolic engineering or the external application of these compounds. In addition, an increase in the expression of the flavanone 3-hydroxylase (F3H) gene in rice was observed, which improved tolerance to drought and UV radiation by boosting non-enzymatic antioxidants kaempferol and quercetin (Figure 3b). These antioxidants help reduce oxidative damage by scavenging ROS and decreasing lipid peroxidation. In comparison to wild plants under similar stress conditions, the transgenic plants exhibited elevated levels of stress-responsive genes (DHN and UVR8), enhanced growth, increased chlorophyll and ion content, and reduced salicylic acid levels [66].

Another study showed that drought stress significantly increases the expression of essential flavonoid biosynthesis genes (TaCHS, TaCHI, TaF3H, TaFNS, TaFLS, TaDFR, TaANS) and boosts the levels of total phenolics, flavonoids, anthocyanins, and schaftoside in the leaves of wheat (*T. aestivum*). The cultivar Chinese Spring has higher overall flavonoid concentrations, while Aikang 58 demonstrates faster gene activation rates. Flavonoids produced in response to drought, particularly anthocyanins, are likely to help reduce oxidative stress through their antioxidant properties, thereby enhancing drought resistance [67].

The research demonstrates that increasing antioxidant flavonoid levels, particularly anthocyanins, in *A. thaliana* by overexpressing genes related to phenolic compound biosynthesis enhances its tolerance to oxidative and drought stress by effectively scavenging reactive oxygen species (ROS) due to their potent radical-scavenging properties. By employing metabolomic, transcriptomic, and genetic methodologies, the scientists discovered that both flavonoid-overproducing and deficient lines of plants rich in anthocyanins had lower ROS levels when under stress. These results highlight the potential benefits of modifying flavonoid biosynthesis in crops to improve their resilience to environmental stress [68].

Another study on drought tolerance in maize seedlings analyzed the drought-resistant mutant doi57, which shows reduced oxidative damage and enhanced growth under water stress compared to the wild-type B73. The authors revealed that flavonoids in doi57 help reduce the accumulation of ROS in guard cells, preserving stomatal opening and photosynthesis while boosting antioxidant capacity, resulting in increased water-use efficiency and improved root-to-shoot ratios [69].

Interestingly, prolonged drought significantly boosts the expression of key genes in the flavonoid pathway in hybrid poplar (*Populus tremula* × *P. alba*), leading to higher production of phenolics, flavonoids, anthocyanins, and carotenoids. The increased antioxidant capacity and activity, along with elevated salicylic acid levels, help mitigate oxidative stress by lowering ROS such as H_2_O_2_ and O_2_^−^, even as photosynthetic parameters decline under drought conditions [70].

**Figure 4:**
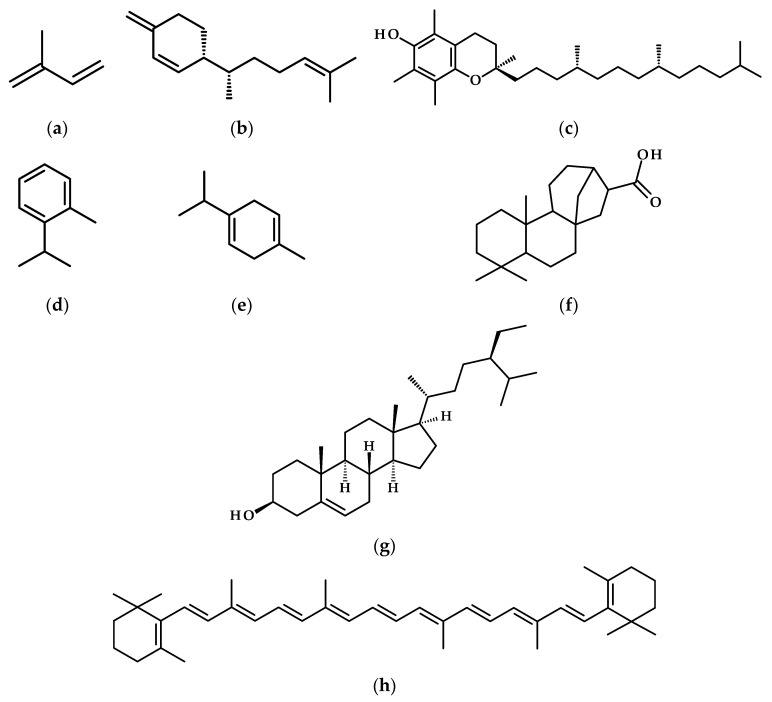
Chemical structures of (**a**) the isoprene unit and various terpenoid derivatives involved in drought stress tolerance, i.e., (**b**) β-sesquiphellandrene, (**c**) α-tocopherol, (**d**) *o*-cymene, (**e**) γ-terpinene, (**f**) kauralexin A1, (**g**) β-sitosterol, and (**h**) β-carotene.

The second group of compounds associated with drought stress includes terpenes, a large class of organic compounds produced by plants. These compounds consist of repeating isoprene units (C_5_H_8_, Figure 4a) and contribute to the distinctive scents and flavors of various herbs, flowers, and fruits. Terpenoids, the oxygen-containing derivatives of terpenes, are vital for plant defense against herbivores and pathogens, and they have widespread applications in industries for fragrances, flavorings, and traditional medicines [71]. Both terpenes and terpenoids are critical for drought stress tolerance in many plants, including herbaceous plants, herbs, and crops.

Drought stress induces specific shifts in terpene and terpenoid levels among various species and genotypes of herbaceous plants, influencing their defense strategies, antioxidant responses, and resource management. In *Tanacetum vulgare*, drought resulted in a decrease in shoot biomass while increasing root terpenoid levels (such as sesquiterpenoids like β-sesquiphellandrene), thereby highlighting belowground chemical defenses [72]. A severe drought in *Rosmarinus officinalis* caused a nine-fold increase in α-tocopherol (a lipid-soluble antioxidant) and an eight-fold rise in oxidized diterpenes (like isorosmanol, derived from carnosic acid), which helped mitigate oxidative damage. Simultaneously, a loss of chlorophyll (85%) reduced light absorption but enhanced the efficiency of antioxidants per absorbed photon [73]. In *Thymus vulgaris*, drought-resistant cultivars (such as Varico3) managed to sustain photosynthesis and water potential longer under stress, alongside persistent increases in vital volatiles like β-phellandrene, *o*-cymene, γ-terpinene, and β-caryophyllene. Conversely, sensitive cultivars (like Wagner) exhibited temporary surges in α-pinene, β-thujene, and ocimene before failing, illustrating a balance between growth and defense [74].

For crop plant studies involving maize, it has been shown that maize roots accumulate terpenoid phytoalexins, zealexins, and kauralexins in response to biotic stressors such as herbivory and fungal infection, as well as abiotic stressors like drought and salinity. Notably, drought-induced levels positively correlate with root-to-shoot ratios across varieties, indicating their role in drought tolerance. Additionally, mutant plants that are deficient in kauralexin production display heightened drought sensitivity, underscoring the significance of these compounds in stress adaptation [75].

A study comparing drought-resistant *Solanum chilense* and drought-sensitive *S. lycopersicum* tomatoes revealed that *S. chilense* upregulated genes GGPPS and HPT1, enhancing α-tocopherol production to mitigate oxidative damage under water stress. Despite similar abscisic acid (ABA) and carotenoid levels, *S. chilense* exhibited lower lipid peroxidation and stomatal conductance, emphasizing α-tocopherol biosynthesis and water conservation as key drought adaptations [76]. A parallel study on Mediterranean long-shelf-life tomatoes in Sicily demonstrated that rewatering after drought increased yields by up to 147% compared to unirrigated plants, while maintaining higher polyphenol and carotenoid levels than fully irrigated plots. Local varieties like ‘Vulcano’ showed exceptional resilience and nutrient retention under stress, highlighting that controlled drought cycles enhance water-use efficiency and preserve antioxidant-rich profiles [77]. Together, these findings emphasize genetic and agronomic strategies, such as α-tocopherol induction and strategic irrigation, to cultivate stress-resilient, nutrient-dense crops in water-scarce regions.

Another study highlights phytosterols’ role in strengthening cell membranes and reducing oxidative damage in water-limited environments. The drought-tolerant (N22) and susceptible (IR64) rice cultivars showed that phytosterols—campesterol, stigmasterol, and β-sitosterol—accumulate under drought stress, with β-sitosterol levels significantly increasing in N22 (364 μg/g FW after 12 days) compared to IR64 (287 μg/g FW). Increased activity of HMG-CoA reductase, a key enzyme in phytosterol biosynthesis, correlated with improved membrane stability and drought tolerance in N22 [78].

Simulated drought (15% polyethylene glycol treatment) in carrots changed carotenoid profiles, with β-carotene and lutein increasing in drought-tolerant cultivars like ‘Kurodagosun’. Upregulation of DcPSY2 and DcLCYB genes drove carotenoid synthesis, while DcNCED genes related to ABA production responded to stress. Cultivar-specific responses indicate that β-carotene and lutein contribute to drought adaptation, guiding the selection of resilient varieties [79].

**Figure 5:**
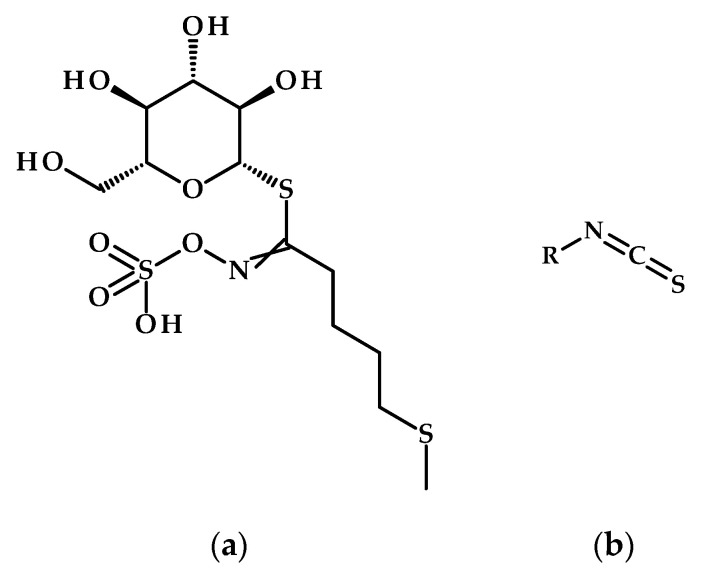
Chemical structures of (**a**) glucoerucin and (**b**) isothiocyanate functional group.

Drought stress in Chinese cabbage (*Brassica rapa* ssp. *pekinensis*) and other *Brassica* species induces oxidative damage, characterized by elevated reactive oxygen species (ROS), malondialdehyde (MDA), and protein carbonylation, alongside reduced relative water content [80,81,82]. Water stress increases glucosinolate content in *B. oleracea*, with leaves accumulating higher levels of aliphatic glucosinolate (e.g., glucoerucin, Figure 5a) compared to roots, which favors indolic and aromatic glucosinolates. Resilient genotypes like broccoli BR1 and cauliflower CV1 exhibit increases of 85.4% and 72.8% in glucosinolate levels in roots and leaves, respectively, under drought conditions [81]. Transcriptome analysis reveals organ-specific gene expression patterns, with roots showing more downregulated genes than leaves, and highlights upregulated transcription factors (bZIP, AP2/ERF) associated with stress adaptation. Glucosinolate degradation products, particularly isothiocyanates (Figure 5b), enhance stomatal closure, reducing water loss and pathogen invasion, underscoring their dual role in drought resilience and defense [80]. Another study also confirmed that glucosinolates in *Brassica* species respond dynamically to abiotic stresses such as salinity, drought, and temperature extremes, modulating biosynthesis pathways and tissue-specific allocation to balance defense and growth. Their degradation products, like isothiocyanates, lessen stress by inducing heat-shock proteins or regulating stomatal closure, while interactions with nutrient deficiencies and biotic stressors highlight crosstalk in signaling networks [82].

Drought stress significantly reduces crop yields by disrupting physiological processes and increasing oxidative damage, making rice, wheat, and maize particularly vulnerable. To enhance resilience, plants utilize phytohormones (e.g., ABA, ethylene) and secondary metabolites (e.g., phenolic compounds, terpenes) through ROS scavenging, stomatal regulation, and antioxidant production. Metabolic engineering or cultivating stress-responsive compounds like anthocyanins and α-tocopherol can strengthen drought tolerance and maintain agricultural productivity in water-scarce conditions.

### 3.3. Temperature Stress

Plants face significant challenges when exposed to temperature extremes—both low and high—that disrupt their physiological and biochemical processes. Like other abiotic stresses, plants attempt to adapt to their environmental conditions by increasing the production of selected metabolites.

One of the review papers [83] identified the aforementioned secondary metabolites related to drought stress, such as proline, glycine betaine, soluble sugars, and polyols, as well as antioxidants (ascorbic acid, glutathione, tocopherols), as critical metabolites that mitigate heat stress in maize by stabilizing membranes, scavenging ROS, and maintaining osmotic balance.

Heat stress from climate change poses a serious threat to wheat production, particularly during the reproductive phases, as it disrupts photosynthesis, accelerates aging, and induces oxidative damage through ROS. To cope, wheat employs antioxidant enzymes (like SOD and CAT) and heat shock proteins (HSPs) that help ensure cellular stability. HSPs, including HSP70 and HSP90, function as molecular chaperones in wheat, preserving the structure of denatured proteins and maintaining cellular integrity under heat stress by preventing protein aggregation and promoting refolding. Their production is initiated by heat stress, with small HSPs (such as HSP20) and HSP100 playing essential roles in combating oxidative stress and facilitating protein disaggregation [84]. HSPs significantly stabilize proteins under various abiotic stresses, including but not limited to heat, drought, salinity, and heavy metal exposure, by reducing misfolding and aggregation. Numerous plant species possess these proteins, among which major HSP families such as HSP70, HSP90, and small heat shock proteins are regulated by heat shock factors (HSFs). Furthermore, HSPs interact with ROS and hormonal pathways, thereby enhancing stress tolerance [85].

Conversely, plants encounter cold stress. To metabolically adapt to cold stress, they accumulate osmolytes (like proline and soluble sugars) and specialized metabolites (such as flavonoids and anthocyanins), and these compounds help stabilize membranes, scavenge ROS, and protect cellular structures. Additionally, carbohydrate metabolism shifts to prioritize trehalose and raffinose synthesis, which serve as cryoprotectants to hinder ice crystal formation, while the breakdown of starch provides energy for adapting to stress [86].

Metabolic changes in *A. thaliana* under heat and cold shocks using gas chromatography–mass spectrometry were also studied [87]. The study identified 81 metabolites and 416 unidentified compounds, revealing that cold shock induced more profound metabolic alterations (311 affected) than heat shock (143 affected). Both stresses elicited similar responses, as most heat-induced changes were also observed during cold shock. These included increases in amino acids (such as pyruvate/oxaloacetate-derived), polyamine precursors, and sugars, implying shared protective mechanisms [87].

**Figure 6:**
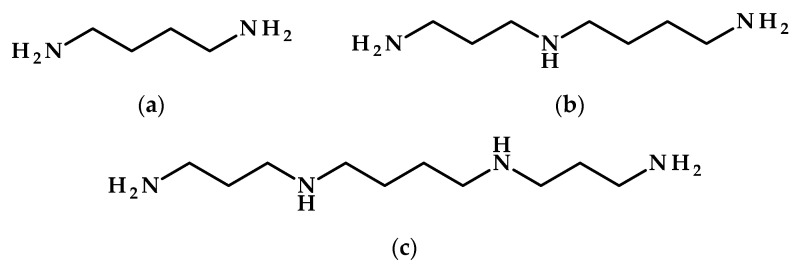
Chemical structures of various polyamines, i.e., (**a**) putrescine, (**b**) spermidine, and (**c**) spermine.

Polyamines—small organic compounds characterized by linear carbon chains and various amine groups, including putrescine, spermidine, and spermine (Figure 6)—play a crucial role in plant stress tolerance, especially in alleviating temperature stress through adaptive metabolic responses [88].

Typically, they play crucial roles in processes such as seed germination, flowering, and fruit development. To manage stress responses, they stabilize cellular structures, scavenge ROS, and interact with hormones and signaling molecules like nitric oxide (NO) [89].

One study examined how exogenous polyamines (spermine and spermidine) help alleviate the effects of heat stress on grain filling in two wheat varieties: the heat-resistant XC6 and the heat-sensitive XC31, under simulated high-temperature conditions. While heat stress reduced yield components and grain weight, the application of these polyamines significantly enhanced antioxidant enzyme activity, diminished oxidative damage, and improved osmotic adjustment (proline, soluble sugars). Spermine was found to be more effective in XC6, whereas spermidine showed better results in XC31. Both polyamines increased endogenous levels of spermine and spermidine, which correlated positively with grain weight, and they also lowered putrescine and stress markers, underscoring their role in maintaining cellular stability under heat stress [90].

A study of cucumber cultivars, specifically the chilling-tolerant Jinchun No. 3 and the sensitive Suyo, regarding chilling tolerance, revealed that spermidine enhances tolerance by inhibiting NADPH oxidase-driven ROS generation, stabilizing membranes, and mitigating oxidative stress, emphasizing its critical role in plant adaptation to low-temperature stress. The tolerant Jinchun No. 3 exhibited significant spermidine accumulation during chilling (3 °C) and rewarming (28 °C/22 °C), which was linked to elevated *S*-adenosyl-Met decarboxylase activity, while the sensitive Suyo showed no such increases, correlating with higher ROS (e.g., hydrogen peroxide) and membrane damage [91].

The untargeted metabolomic analysis of tomato pollen across three developmental stages (polarized microspores, bicellular pollen, and mature pollen) under control conditions (22 °C) and short-term heat stress (38 °C for 2 h) revealed stage-specific metabolic shifts and stress responses. Under control conditions, young microspores accumulated alkaloids (e.g., α-tomatine and β-tomatine) and conjugated polyamines (e.g., coumaroyl/caffeoyl spermidine), while mature pollen primarily stored flavonoids (e.g., kaempferol dihexoside), suggesting roles in defense and stress protection during development. Heat stress induced a significant two-fold increase in total flavonoids in polarized microspores, likely serving as an antioxidant response to reduce oxidative damage, but did not change polyamine or alkaloid levels, highlighting the role of flavonoids in early thermotolerance [92].

In conclusion, plants employ a multifaceted metabolic strategy to combat temperature stress, utilizing heat shock proteins (HSPs), osmolytes, antioxidants, and polyamines to stabilize cellular structures, mitigate oxidative damage, and maintain homeostasis under both heat and cold extremes. Understanding these metabolic and molecular pathways provides critical insights for developing climate-resilient crops to safeguard global food security amid rising temperature variability.

### 3.4. Marker-Assisted Selection in Plant Breeding for Stress Tolerance

Marker-assisted selection (MAS) is a biotechnology-driven approach in plant breeding that uses molecular markers linked to desirable traits to accelerate the development of stress-tolerant crops. MAS has transformed plant breeding by enabling the precise incorporation of stress-tolerant traits into elite crop varieties. By utilizing molecular markers associated with quantitative trait loci (QTLs) or specific genes, breeders can effectively select favorable genotypes while avoiding labor-intensive phenotypic screening [93,94].

In cereals like rice, MAS has successfully introduced salinity tolerance genes such as Saltol, a major QTL on chromosome 1, into high-yielding cultivars like IR64, enhancing salt resilience at the seedling stage [95]. Similarly, the pyramiding of multiple disease resistance genes—Pi9 and Pb1 for blast, and Xa4, xa13, and Xa21 for bacterial blight—has resulted in rice lines with durable resistance, as demonstrated in BRRI dhan48, which sustained yield under pathogen pressure [94].

For abiotic stresses like drought and heat, MAS identifies QTLs related to root architecture, canopy temperature depression, and photosynthetic efficiency [69,81]. In maize, heat tolerance is linked to QTLs that control pollen viability and grain filling. Genomic studies have identified candidate genes, including ZmHSP101 and antioxidant enzymes, that help mitigate oxidative damage [83]. Omics techniques (including genomics, transcriptomics, and proteomics) enhance MAS by elucidating stress-responsive pathways, such as salicylic acid signaling for blast resistance and osmolyte accumulation for drought adaptation [95,96].

Despite notable successes, challenges remain, including limitations in marker polymorphism, epistatic interactions, and the polygenic nature of stress tolerance [93,94]. Advances in high-throughput genotyping (SNP arrays, genotyping-by-sequencing) and genome editing (CRISPR-Cas9) now allow for precise manipulation of target genes, facilitating faster trait introgression [80]. Integrating MAS with speed breeding and genomic selection improves prediction accuracy for complex traits [95].

Future efforts will focus on combining multi-omics data, utilizing haplotype-based breeding, and engaging participatory methods to address region-specific stresses [95,96]. By linking molecular insights with agronomic practices, MAS remains vital in developing climate-resilient crops, thereby ensuring food security in the face of increasing environmental challenges [93,94].

## 4. Integration of Metabolomics with Other “Omics”

Metabolomics offers direct insight into the biochemical state of a plant under stress, capturing changes in primary and secondary metabolites. These small molecules reflect the real-time physiological adjustments that occur in response to environmental stress, making metabolomics a valuable measure of plant health and stress tolerance. Metabolites represent the end products of gene expression and protein activity, thus providing a direct link to plant phenotypes under stress.

Integrating metabolomics with other “omics” technologies such as genomics, transcriptomics, and proteomics, gives a comprehensive, system-level understanding of how crops respond to abiotic stresses, including drought, salinity, temperature extremes, or nutrient deficiencies. This holistic approach is essential for developing stress-tolerant crop varieties and optimizing breeding and biotechnology strategies.

### 4.1. The Integration of Genomics and Metabolomics

Genomics and metabolomics are complementary approaches that, when integrated, provide a comprehensive understanding of how plants respond and adapt to various adverse conditions. The use of genomics enables the identification and characterization of genes and genetic loci (e.g., quantitative trait loci, QTL) associated with abiotic stress tolerance [97]. Advances in sequencing techniques have facilitated genome-wide expression profiling, which helps to localize stress-responsive genes and regulatory networks. Furthermore, marker-assisted selection (MAS) and genomic selection are employed to breed plants with improved stress tolerance by targeting desirable alleles [98]. Additionally, metabolomics allows us to profile complete sets of metabolites in plants, revealing biochemical changes under stress. It is possible to identify key metabolites (e.g., proline, sugars, and antioxidants) that accumulate in response to stress and contribute to tolerance. Furthermore, metabolomics can link gene function to phenotype, demonstrating how genetic variation affects metabolic pathways and stress adaptation [99].

Combining genomics and metabolomics enables researchers to connect specific genes or alleles to metabolic traits that confer stress tolerance. This integration aids in identifying regulatory and metabolic networks that underlie stress responses, providing a system-level view of plant adaptation [99]. For example, metabolomics studies have shown that drought-stressed plants accumulate specific amino acids and sugars. Additional genomic applications can pinpoint genes that govern these metabolic changes [100]. Integrated approaches thus facilitate the identification of molecular markers and metabolic signatures for use in precision breeding and genetic engineering of stress-tolerant crops. They also lay the groundwork for systems biology approaches, enabling predictive modeling of crop stress responses and contributing to targeted breeding and biotechnological strategies for developing resistant crop varieties.

### 4.2. The Integration of Transcriptomics and Metabolomics

Transcriptomics profiles genome-wide gene expression changes in response to abiotic stress by identifying differentially expressed genes (DEGs) and regulatory networks. The application of transcriptomics enables the identification of key transcription factors, signaling pathways, and stress-responsive genes that coordinate physiological and metabolic adjustments [101]. Metabolomics, on the other hand, quantifies dynamic changes in plant metabolites under adverse conditions, capturing the biochemical outcomes of gene regulation.

Combining transcriptomic and metabolomic data reveals how gene expression changes translate into metabolic reprogramming, demonstrating crosstalk between signaling pathways and metabolic networks under stress [101]. Furthermore, integrated studies have shown that genes involved in sugar and amino acid metabolism are upregulated under drought or nutrient stress, leading to the accumulation of compatible solutes and antioxidants that protect plant cells [101]. Comparative analyses between stress-tolerant and stress-sensitive genotypes can uncover unique transcriptomic and metabolic signatures associated with resistance, as observed in studies of barley and maize. This common approach helps identify candidate genes and metabolites that serve as biomarkers for breeding stress-tolerant varieties [102]. In maize, integrated transcriptomic and metabolomic profiling under low-phosphorus stress conditions revealed that immune regulation and pathways related to carbon, fatty acid, and amino acid metabolism are activated, linking specific gene expression changes to metabolic changes that support yield under stress. In medicinal and wild crops, this integration has clarified how drought or heavy metal stress leads to coordinated increases in flavonoids, polyamines, soluble sugars, and glutathione compounds essential for osmotic regulation and antioxidant defense.

Therefore, the integration of transcriptomics and metabolomics offers a comprehensive perspective on how crops perceive, process, and respond to abiotic stress at both molecular and biochemical levels. This collaboration facilitates the identification of key regulatory genes, pathways, and metabolites, speeding up the development of crops with improved stress tolerance.

### 4.3. The Integration of Proteomics and Metabolomics

The use of proteomics enables the study of changes in protein abundance, modifications, and interactions under stress conditions. This technique identifies stress-responsive proteins, including enzymes, transporters, and regulatory proteins that mediate mechanisms of cellular adaptation and defense [103]. Proteomic analysis reveals how protein expression and post-translational modifications (PTMs) are altered during stress, providing insight into the dynamic regulation of stress tolerance pathways [104]. Metabolomics profiles small-molecule metabolites generated by cellular processes, capturing the biochemical outcomes of protein activity and regulation. It identifies key metabolites, such as osmoprotectants, antioxidants, and signaling molecules, that accumulate in response to stress, directly contributing to plant survival and adaptation [105].

The integration of proteomics and metabolomics uncovers molecular networks and pathways that regulate stress resistance, linking protein function to metabolic changes. This approach enables the identification of novel proteins and metabolites that are crucial for stress adaptation and cannot be revealed by single-omics studies alone. The combined approach offers a systems biology perspective, connecting gene expression, protein activity, and metabolic outcomes to the phenotypes of stressed plants [106]. By recognizing key molecular players, breeders can select traits associated with both protein and metabolite markers, enhancing the efficiency of crop improvement programs. In turn, understanding the synergistic roles of proteins and metabolites aids in developing crops that utilize nutrients more efficiently and withstand environmental challenges, contributing to sustainable agriculture.

### 4.4. Benefits and Challenges of Multi-Omics Integration

Integrating data from multiple omics disciplines—genomics, transcriptomics, proteomics, and metabolomics—enables a holistic and more precise understanding of plant responses to abiotic stresses. This comprehensive approach reveals the complex regulatory networks and signaling pathways underlying stress perception, signal transduction, and metabolic adaptation. Unlike single-omics methods, multi-omics analyses uncover genes, proteins, and metabolites—such as osmoprotectants and antioxidants—that may remain undetected in less comprehensive studies. For instance, integration of transcriptomic and metabolomic data in rice has elucidated both ABA-dependent and ABA-independent pathways that regulate drought responses [107].

Multi-omics data integration facilitates mapping hierarchical signaling networks from genotype to phenotype, providing insights into the dynamic and tissue-specific nature of plant stress responses. Time-course analyses, combined with spatial profiling (e.g., comparing roots vs. leaves), allow researchers to track transitions from early stress perception to long-term acclimation. This knowledge forms the foundation for designing targeted genetic engineering strategies and precise metabolic interventions to enhance stress tolerance [108,109].

The practical applications of multi-omics integration are significant, particularly in the context of precision crop improvement. Multi-omics facilitates the identification of robust biomarkers and quantitative trait loci (QTLs) associated with resistance traits, thus improving the efficiency of marker-assisted selection (MAS) and genomic selection (GS) [110]. For example, combining metabolomic profiles with genomic data has enhanced the accuracy of predicting drought and heat tolerance in crops like rice and wheat [111,112]. The identification of key transcription factors, metabolic enzymes, and transporters involved in hormone signaling and oxidative stress mitigation has further enabled targeted genetic modifications, such as the manipulation of polyamine biosynthesis in salt-stressed rice [113].

Moreover, integrated omics analyses are helping to uncover epigenetic and metabolic memory mechanisms, allowing plants to “remember” prior to stress exposures and mount faster responses upon subsequent encounters. These insights enable genotype-specific crop management strategies tailored to particular environmental conditions and stress factors.

#### Methodological Limitations of Multi-Omics Integration

Despite its promise, the implementation of multi-omics approaches in plant stress biology faces substantial methodological, technical, and computational hurdles [114]. A primary challenge lies in the complexity and heterogeneity of the data. Omics layers differ in their nature, scale, and data formats—requiring sophisticated preprocessing steps such as normalization, scaling, and alignment to ensure meaningful integration [115,116].

Each omics platform demands unique sample preparation and preservation protocols—flash freezing for metabolomics, RNA stabilization for transcriptomics—complicating standard workflows and increasing the risk of data loss or bias [117]. Additionally, incomplete coverage of biological molecules remains an issue: current metabolomics techniques (e.g., LC-MS, GC-MS, and NMR) may miss unstable or low-abundance compounds, while proteomics struggles with detecting isoforms and post-translational modifications [118].

High-throughput omics technologies are prone to technical noise and missing values, particularly in large-scale field studies. Moreover, integrating disparate data types—such as transcript levels, protein abundances, and metabolite concentrations—into a coherent biological narrative remains difficult due to differences in data scales and measurement units [118].

Computational demands are another major barrier. Most tools currently available are tailored to individual omics types and do not support true cross-platform integration [117]. Analyzing multi-omics data often requires specialized expertise, high-performance computing infrastructure, and mastery of complex methods such as machine learning, Bayesian inference, or network-based approaches [113].

Lastly, the cost of multi-omics studies—including sequencing, proteomic, and metabolomic analyses—remains prohibitively high, limiting their widespread use in commercial breeding programs. Even when data are successfully integrated, interpreting findings within a relevant biological context requires considerable manual effort and domain expertise, especially given the nonlinear and dynamic nature of biological systems [119,120].

## 5. Integrative Tools and Platforms for Multi-Omics Data Analysis

Multi-omics analyses, encompassing genomics, transcriptomics, proteomics, and metabolomics, offer a comprehensive view of plant responses to environmental stimuli. However, leveraging these datasets requires advanced bioinformatics tools capable of managing diverse data formats, integrating heterogeneous data types, and supporting their visualization and interpretation [121,122]. Proper preprocessing of omics data is essential for reliable downstream analysis.

In metabolomics, one of the key analytical platforms is MetaboAnalyst “https://www.metaboanalyst.ca/” (accessed on 21 May 2025), which provides functions for normalization, scaling, imputation of missing values, and batch effect correction. For transcriptomics, R packages such as DESeq2 and edgeR are widely used to detect differential gene expression from RNA-seq data [123]. Proteomic data, typically derived from mass spectrometry, are commonly processed using tools like ProteoWizard “https://proteowizard.sourceforge.io/” (accessed on 21 May 2025) and MaxQuant “https://maxquant.org/maxquant/” (accessed on 21 May 2025), which facilitate peptide identification and quantification

Statistical and multivariate methods play a central role in handling high-dimensional omics datasets by enabling dimensionality reduction and the identification of latent structures. SIMCA “https://www.sartorius.com/en” (accessed on 21 May 2025), a commercial software package widely used in metabolomics, supports techniques such as PCA and PLS-DA. Alternatively, programming environments like R and Python offer extensive libraries—including mixOmics “https://mixomics.org/” (accessed on 21 May 2025)—that facilitate data exploration, classification, and the application of machine learning algorithms.

Data integration across different omics layers is a crucial step for obtaining a system-level understanding of the complex biological mechanisms underlying abiotic stress responses. The DIABLO module within the mixOmics R package allows for supervised integration of transcriptomic, proteomic, and metabolomic data [124]. Other tools, such as iClusterPlus and MOFA+ [125], employ latent variable models to uncover shared patterns and molecular subtypes across omics datasets.

Cytoscape, “https://cytoscape.org/” (accessed on 21 May 2025) in combination with plugins like MetScape and ClueGO, facilitates the integration and visualization of omics data as molecular interaction networks and supports functional enrichment analysis. For biological interpretation, omics data must be mapped to known pathways and gene functions. Tools such as KEGG “https://www.genome.jp/kegg/” (accessed on 21 May 2025) provide curated knowledge bases of metabolic pathways and enable data visualization within this context. MapMan “https://mapman.gabipd.org/mapman” (accessed on 21 May 2025), a plant-specific platform, supports the analysis of transcriptomic and metabolomic data within functional categories [126].

Furthermore, protein–protein interaction (PPI) networks derived from platforms such as STRING “https://string-db.org/” (accessed on 21 May 2025) and GeneMANIA “https://genemania.org/” (accessed on 21 May 2025) aid in the identification of co-regulated protein modules involved in stress responses. Machine learning approaches are increasingly applied in multi-omics studies for stress response classification, phenotype prediction, and biomarker discovery. Frameworks like TensorFlow 2.16.1 and PyTorch 1.13 enable deep learning-based modeling of complex relationships, while algorithms such as Random Forest and Support Vector Machine (SVM)—available in both R and Python—are effective for classification and feature selection in regulatory network analysis [127,128].

Access to high-quality reference databases and repositories is essential for accurate data annotation and comparative analyses. The Plant Metabolic Network (PMN) offers comprehensive information on metabolic pathways across various plant species. Databases such as Phytometasyn, MetaCyc, and PlantCyc specialize in plant-specific metabolite and biosynthetic pathway data. Public repositories like Gene Expression Omnibus (GEO), EMBL-EBI Expression Atlas, and MetaboLights contain extensive transcriptomic and metabolomic datasets generated under stress conditions.

Finally, the increasing complexity of multi-omics data necessitates sophisticated computational approaches and system-level integration [129]. Similarity- and correlation-based methods enable the classification and identification of co-regulated molecular entities. Network-based and Bayesian inference methods facilitate interaction modeling and probabilistic data integration [129,130]. Systems omics integration (MOI) tools allow the analysis of metabolic pathways, regulatory networks, and the identification of key molecular regulators driving phenotypic outcomes, making them indispensable in advancing our understanding of abiotic stress tolerance in plants [120].

## 6. Future and Practical Applications

Metabolomics, as an integrated and high-resolution biological analysis tool, offers transformative potential for enhancing crop tolerance to abiotic stress. Its numerous practical and forward-looking applications range from modern breeding strategies to precise metabolic engineering. The aim is to develop crop varieties capable of maintaining high productivity despite increasingly unfavorable environmental conditions. By decoding the complex metabolic responses of plants to abiotic stressors, metabolomics enables scientists to integrate knowledge of stress physiology with practical crop improvement strategies [131]. Thus, its future in agricultural sciences lies not only in expanding our understanding of stress tolerance mechanisms but also in implementing this knowledge through specific, applicable solutions under field conditions.

### 6.1. Metabolomics-Assisted Breeding

One of the most promising applications of metabolomics in agricultural practice is its integration with breeding programs aimed at increasing plant resistance to abiotic stress. Unlike genomics or transcriptomics, which provide information on the potential responses of an organism, metabolomics captures actual, dynamic physiological changes, offering a direct reflection of phenotypic expression [42]. Profiling metabolites associated with stress responses—such as osmoprotectants (e.g., proline, glycine betaine), antioxidants (ascorbate, glutathione), or secondary metabolites (flavonoids, alkaloids)—enables the identification of metabolic markers closely linked to resistance phenotypes [132]. These markers can serve breeders as effective selection tools. For example, elevated levels of proline or glycine betaine may indicate drought tolerance [133].

Importantly, the use of metabolic markers—including metabolite quantitative trait loci (mQTL) and metabolome-wide association studies (MWASs)—allows for more precise selection of elite germplasm than traditional genetic markers, which are often sensitive to environmental variability [134,135]. Metabolomics-assisted breeding accelerates the selection process by providing a biochemical snapshot of plant stress responses and facilitating the selection of genotypes capable of maintaining metabolic homeostasis under challenging conditions [131]. Furthermore, this approach helps balance trade-offs between stress tolerance and crop quality, as demonstrated by studies showing correlations between metabolite profiles and traits related to fruit quality and stress resistance [136].

### 6.2. Metabolic Engineering

Modern genetic engineering, supported by data from metabolomic analyses, is becoming an increasingly precise tool for developing crops with enhanced resistance to abiotic stress. Metabolomics, by revealing changes in metabolic pathways in response to stress, identifies specific targets for genetic modification, such as pathways involved in antioxidant biosynthesis, osmolyte accumulation, or regulation of energy metabolism [137,138]. These insights form the basis for targeted gene editing aimed at strengthening plants’ natural defense mechanisms.

Advances in genome editing technologies—particularly CRISPR/Cas9—open new possibilities in the field of so-called metabolic editing, guided by metabolomic insights [139]. By identifying genes that control key pathways related to stress tolerance, it becomes possible to precisely modify metabolite profiles to achieve desired phenotypic traits [140]. Metabolomics plays a dual role in this process—not only guiding the identification of editing targets, but also serving as a validation tool, enabling the monitoring of modification effects and minimizing the risk of unintended metabolic consequences [141].

In addition, emerging synthetic biology approaches enable the design of artificial metabolic circuits that enhance stress resistance without compromising yield [142,143]. The integration of synthetic pathways with native plant metabolism may, in the future, enable the development of crop varieties capable of thriving under extreme climatic conditions.

### 6.3. Understanding Stress Memory and Epigenetic Effects

Metabolomics-assisted approaches open new perspectives not only for improving plant resistance to stress but also for studying so-called stress memory and epigenetic modifications at the metabolic level. Understanding how plants “remember” previous stress episodes through lasting changes in metabolism and epigenetic regulation may play a key role in developing breeding strategies aimed at enhancing the long-term tolerance of crops to abiotic stress [144,145]. This approach supports the development of plants with durable resistance that are better adapted to changing environmental conditions.

Metabolomics also has practical applications in improving crop quality—not only by enhancing stress resistance but also by regulating secondary metabolites responsible for nutritional and organoleptic properties [146]. Metabolite profiling under water stress conditions has shown that changes in flavonoid and phenylpropanoid content are often cultivar-specific and have a significant impact on fruit quality [147]. These findings indicate the potential for using metabolomic data in agronomic decision-making—such as cultivar selection, grafting, or irrigation management—to maintain high crop quality even under stress conditions [148].

Ultimately, integrating metabolomics with modern breeding and crop management strategies contributes to enhancing global food security. It enables the development of plants that are not only resistant to climate change and capable of maintaining stable yields but also retain quality traits essential for consumer health and the commercial value of crops [131].

## 7. Conclusions

This study highlights the crucial role of metabolomics in deciphering the biochemical complexity of plant responses to abiotic stress. By integrating both targeted and untargeted metabolomic analyses across various crop species and stress conditions, we have identified conserved and stress-specific metabolic adjustments that enhance resilience. Key metabolites, such as osmoprotectants, antioxidants, and signaling molecules, have emerged as vital components of adaptive responses, while novel biomarkers and metabolic hubs provide actionable targets for genetic improvement. These insights bridge the gap between molecular phenotype and stress tolerance, paving the way for precision breeding and metabolic engineering strategies. Ultimately, our findings affirm metabolomics as a cornerstone of next-generation crop improvement programs, with profound implications for sustainable agriculture in an era of climatic uncertainty.

## Figures and Tables

**Table 1:** Method comparison.

Feature	GC-MS	LC-MS	NMR Spectroscopy
Sensitivity	High	Very high	Moderate
Reproducibility	High	Moderate–high	Very high
Sample preparation	Requires derivatization	No derivatization needed	Minimal, especially for HR-MAS
Metabolite coverage	Volatile, polar metabolites	Broad (polar and non-polar)	Limited, mostly abundant metabolites
Quantification	Relative or absolute (with standards)	Relative or absolute (with standards)	Absolute without standards
Structural elucidation	Limited	Limited to fragmentation data	Strong (direct molecular structure)
Destructive analysis	Yes	Yes	No
Throughput	Moderate	High	Moderate
Cost (instrument and maintenance)	Moderate–high	High	Very high
Common applications	Sugars, amino acids, and organic acids	Secondary metabolites, lipids, phenolics	Structural ID, metabolite fingerprinting

Abbreviations: GC-MS—gas chromatography–mass spectrometry; LC-MS—liquid chromatography–mass spectrometry; NMR—nuclear magnetic resonance; HR-MAS—high-resolution magic angle spinning.

**Table 2:** Workflow for plant sample preparation in metabolomics.

Step	Purpose	Methods/Techniques	Practical Notes
Experimental Design	Ensure biological relevance and statistical robustness	Define replicates, randomization, and sample size	Standardize environmental/growth conditions
2. Sample Collection	Preserve metabolic state at harvest	Rapid harvesting, consistent timing	Avoid contamination; handle with gloves/tools
3. Quenching	Halt metabolic activity immediately	Flash-freezing in liquid nitrogen, cold solvents (methanol, dry ice–ethanol)	Store at −80 °C; transport on dry ice
4. Homogenization	Disrupt tissue for uniform extraction	Cryogenic grinding (mortar and pestle or bead beater)	Prevent thawing; use precooled tools
5. Metabolite Extraction	Isolate metabolites from the plant matrix	Solvent systems (methanol:water, chloroform:methanol:water, MTBE, etc.)	Select a solvent based on target metabolites and platform
6. Clean-Up	Remove solids and reduce matrix effects	Centrifugation, filtration (0.2 μm), Solid Phase Extraction	Avoid contamination, work on ice
7. Derivatization (GC-MS only)	Improve volatility and stability	Oximation + silylation (e.g., MSTFA)	Required for GC-MS; not needed for LC-MS or NMR
8. Reconstitution	Prepare a sample for analysis	Reconstitute in LC/NMR-compatible solvents (e.g., acetonitrile:water, D_2_O)	Use appropriate internal standards
9. QC Implementation	Ensure analytical reproducibility	Use pooled QC samples, internal standards, and randomized injection order	Monitor instrument drift and variability
10. Storage	Maintain sample integrity	Freeze at −80 °C; minimize freeze-thaw cycles	Aliquot samples for repeatability

Abbreviations: GC-MS—gas chromatography–mass spectrometry; LC-MS—liquid chromatography–mass spectrometry; NMR—nuclear magnetic resonance; MTBE—Methyl *tert*-Butyl Ether; MSTFA—*N*-Methyl-*N*-(trimethylsilyl)trifluoroacetamide.

## Data Availability

No new data were created or analyzed in this study. Data sharing does not apply to this article.

## Abbreviations

The following abbreviations are used in this manuscript:

ABA	Abscisic acid
APX	Ascorbate Peroxidase
BADH	Betaine-Aldehyde Dehydrogenase
BR	Brassinosteroids
Cas9	CRISPR-associated protein 9
CAT	Catalase
CE-MS	Capillary electrophoresis–mass spectrometry
CRISPR	Clustered Regularly Interspaced Short Palindromic Repeats
DEGs	Differentially expressed genes
DIA	Data-independent acquisition
EI	Electron ionization
ET	Ethylene
F3H	Flavanone 3-hydroxylase
FT-IR	Fourier transform infrared spectroscopy
GC-MS	Gas chromatography–mass spectrometry
GEO	Gene Expression Omnibus
GS	Genomic selection
HR-MAS	High-resolution magic angle spinning
HR-MS	High-Resolution mass spectrometry
HSFs	Heat shock factors
HSPs	Heat shock proteins
IMS-MS	Ion mobility mass spectrometry
JA	Jasmonic acid
KEGG	Kyoto Encyclopedia of Genes and Genomes
LC-MS	Liquid chromatography–mass spectrometry
MAS	Marker-assisted selection
MDA	Malondialdehyde
MOI	Multi-omics integration
MSI	Metabolomics Standards Initiative
MTBE	Methyl *tert*-Butyl Ether
MSTFA	*N*-Methyl-*N*-(trimethylsilyl)trifluoroacetamide
MWAS	Metabolome-wide association study
NIST	National Institute of Standards and Technology
NMR	Nuclear magnetic resonance
NO	Nitric oxide
PCA	Principal component analysis
PLS-DA	Partial least squares discriminant analysis
PMN	Plant Metabolic Network
PPI	Protein–protein interaction
PTMs	Post-translational modifications
QC	Quality control
QTL	Quantitative trait loci
ROS	Reactive oxygen species
SA	Salicylic acid
SL	Strigolactones
SNP	Single nucleotide polymorphism
SOD	Superoxide Dismutase
SOPs	Standard Operating Procedures
SVM	Support vector machine
T6P	Trehalose-6-phosphate
TSP	Trimethylsilylpropanoic acid
